# Dr. Bouttatz's Cases of Catalepsy and Epilepsy

**Published:** 1801-11

**Authors:** F. Bouttatz

**Affiliations:** Charlotte Street, Fitzroy Square


### To the Editors of the Medical and Physical Journal.

GENTLEMEN,

YOU will oblige me by inserting the following Cases of Catalepsy and Epilepsy in your very useful Journal.

I am, &c.

*Charlotte Street, Fitzroy Square,*

*October* 5, 1801.

F. BOUTTATZ.

---

I was requested to visit a girl, about nine years of age, who had been afflicted with violent periodical fits for the course of two years. According to the account I received from her parents, these fits were always preceded by a total stiffness in all her limbs, her eyes at the same time remaining fixed to one point, whilst she continued to stand in the very spot where the fit had first seized her, without moving either hand or foot. In the commencement of the disorder, the fits continued only a few minutes, but afterwards increased; every successive fit was stronger and more lasting. I must also observe, she would then drop down and instantly fall asleep.

About six months before I was consulted, additional symptoms were observed. The child now dropped to the ground, as soon as the stiffness went off, and was then seized with the most dreadful convulsions; tore all her clothes, and kicked most violently, whilst she foamed, sighed, screamed, and clenched her thumbs close within her hands. She had been seized with her last fit about the end of August, a fortnight before I saw her.

From this description the disease appeared to be catalepsy, attended with epilepsy. Upon examining the child, I found her pale and emaciated; her belly prodigiously inflated, her pulse low and irregular. The parents were in perfect health, and could not recollect a similar case ever occurring in their family; therefore, the disease could not be hereditary. The only ground upon which I could form any supposition as to the cause of these fits, was, the extreme poverty of her parents, who lived almost entirely upon potatoes.

I enquired whether they had never discovered in the child any symptoms of worms, or whether they had not already employed some other physician? Upon which I was told by the mother, that she had expended, for the recovery of her child, more than her circumstances could properly afford; but having after all, perceived not the least prospect of amendment, she had had refolved to leave the difeafe entirely to Nature, and that fhe had never difcovered the leaft appearance of worms. When I reflected upon all thefe fymptoms, and particularly the inflated ftate of the abdomen, I refolved to adminifter the moft gentle purgatives, being apprehenfive of increafing the irritable ftate of the child's body, and therefore prefcribed fmall dofes of calomel, with a fmall quantity of rhubarb. When I vifited my patient the day after fhe had taken the purgative, I found that it had brought away a quantity of flime, and was told by herfelf, that the medicine fhe had taken agreed extremely well with her. I then defired her parents to fend for me as foon as her fits fhould return.

On the third day I ordered her to take fifteen grains of crem. tart. and the fame quantity of magnefia, every day; this medicine was continued for five days, and produced eafy ftools. Having fome fufpicion that her difeafe was occafioned by worms, I prefcribed femen fantonici with honey. After continuing this medicine for four days, I ordered her to take a purge of calomel and jalap: The evacuations which enfued, confirmed my fufpicion; a great number of living and dead afcarides, mixed with flime, being difcharged; after which the child felt herfelf greatly relieved.

In the evening of Sept. 29, I was informed, however, that fhe had been again feized with a fit: I found her quite ftiff, leaning againft a chair, with her eyes immoveably fixed to one point. This ftate of total torpor lafted about fifteen minutes, when fhe fuddenly dropped down, and fell into violent convulfions. She kicked the chair away, tore her clothes and hair, fcreamed, wept, and held her thumbs fo firmly enclofed in her hands, that they could not be difengaged. This fit lafted longer than the firft, and terminated in a profound fleep, from which the child awoke in a ftate of extreme fatigue, without having the leaft recollection of the fituation in which fhe had been.

The parents affured me, that this had been the leaft violent fit which fhe had had within the laft fix months. Vague as the affertions of people of that clafs generally are, this information made me conclude ftill more confidently, that the child's difeafe was occafioned by nothing elfe but worms. I ordered the worm medicines to be continued, prefcribing, at the fame time, the vinous extract of abfynthium, of which the patient took a wine glafs full every morning; at the fame time mild purgatives were occafionally adminiftered. Towards the end of October the paroxyfm returned, but, as I clearly obferved, with lefs violence than the time before; the fecond, i. e. the epileptic fit, lafting only twenty minutes.

Her

Her convulsions likewise, were less violent, and her sleep of shorter duration than heretofore. This inspired me with the most sanguine hope of being able to effect a radical cure.

Observing that the fits returned regularly once a month, I added, to the use of the calomel, the bark and limatura ferri, ordering, at the same time, the abovementioned extract of absynthium to be continued. It is necessary to observe here, that the age of the girl did not permit me to hope, that the commencement of the monthly evacuations might put an end to her disease.

Perceiving that the bark did not agree with my patient, as it produced too frequent stools, I added a small quantity of opium. After she had continued the use of these medicines eight days, I directed her to take a purgative, when a great quantity of ascarides and slime were discharged by stool. I recommended exercise and gentle frictions of the abdomen: The belly became softer, and visibly decreased. On the last day of October, one day later than usual, the paroxysm returned, but to my greatest astonishment, was confined only to a fit of the catalepsy, no epileptical symptoms being perceived: This inspired me with fresh hope of being able to restore the child entirely. I prescribed bark and valerian, limatura martis, and orange peel, whilst I made her continue the use of extract. absinth. and had the satisfaction to perceive, that the child increased in strength and health.

These medicines were continued a whole month, and the 3d of December, about four days later than usual, the child had again a cataleptic fit; but no signs of epilepsy appeared. On the 5th of the same month I prescribed the usual purgative, which carried off whole lumps of ascarides, mixed with a great quantity of slime.

On the following day, she again took the bark mixed with valerian, limatura martis, and semen santonici.

On January 10th, about eight days later than usual, she was again seized with a fit of catalepsy, which however lasted only five minutes. The short duration of this paroxysm gave me hopes of a speedy termination of the disease. The last mentioned medicines were continued, and the catalepsy was at length totally subdued. In order to stimulate the parents to bestow the most unremitted care and attention on the child, I observed to them, that, favorable as appearances might be, they must continue to give her every dietetic and medical assistance in their power for some time longer, as relapses were extremely frequent in diseases of that kind, and that those relapses in general proved incurable. This injunction produced the desired effect; I continued to attend the patient for a few months longer, longer, efpecially at the time when the periodical return of the difeafe might be expected. I then fuffered her gradually to difcontinue the ufe of the medicines, and, after four months, had the pleafure of feeing her completely cured.

---

I was confulted by a friend refpecting a farmer's fon, a youth about eighteen years old, of a robuft conftitution, but who for fix months paft had been frequently feized with epileptic fits. The parents of the young man, as is generally the cafe with common people, kept the difeafe of their fon as fecret as poffible.

On examining him, I found his complexion very pale, and his belly uncommonly protuberant and hard. Although his ftrength had not been much impaired, yet he was indolent and unfit for any work. His fits were not periodical, but generally lafted very long. The farmer, being anxious for the recovery of his fon's health, brought him to town, and put him entirely under my care.

I prefcribed, in the firft place, ipecac. and tart. emet. in order to clear the primæ viæ. The emetic operated feveral times, both upward and by ftool. The next day I ordered him to take a purgative of jalap and calomel, that produced feveral evacuations, in which however there was not the leaft appearance of worms. Three days after, about ten o'clock at night, I was informed that he had been feized with a fit, which I perceived at firft fight to be completely epileptical.

His convulfions were uncommonly violent, and I was informed by his friends that this fit was the moft dreadful he had ever had. His mouth was covered with froth, whilft he kicked with the greateft vehemence his cloaths to pieces. The people under whofe care he was, were firmly convinced that his life could not be faved, and fent for a clergyman in order to prepare him for his diffolution. But the patient paid not the leaft attention to the clergyman, for when he attempted to adminifter the facrament, ftruck the cup out of his hand. I was obliged to be a paffive fpectator of this fcene, as I could neither adminifter any affiftance to the patient, nor prevail on the byftanders to leave him at reft.

The convulfions lafted an hour, the patient then fell into a doze, from which he awoke after a confiderable time, extremely exhaufted, and totally ignorant of what had happened to him. The next day he complained only of extreme weaknefs and faintnefs, and told me that he often felt a violent craving for food, attended with an itching in the nofe. This led me ftrongly to fufpect that his difeafe was occafioned by worms, for for which reafon I prefcribed fem. fant. with honey; ordering him to take every day as much as he could bear. After he had taken this medicine eight days, I gave him another purgative of jalap and calomel, and was aftonifhed, upon vifiting him the next day, to find that a whole pot full of afcarides had been difcharged. The patient informed me that he felt himfelf greatly relieved, and was now totally free from that heavinefs with which he had been oppreffed. I made him continue his medicine, ordering him, at the fame time, to drink every morning a glafs of infus. abfynth. diluted with water. He now vifibly recovered his ftrength, and his appetite encreafed. After a week I prefcribed another purgative, which carried off a great quantity of afcarides as well as a great deal of flime. I continued the medicine two months longer, which had been fo fuccefsfully applied, and not the leaft trace of epilepfy was to be obferved. The young man grew frefh, ruddy, ftrong, and extremely lively.

